# Dynamic Nanohybrid-Polysaccharide Hydrogels for Soft Wearable Strain Sensing

**DOI:** 10.3390/s21113574

**Published:** 2021-05-21

**Authors:** Pejman Heidarian, Hossein Yousefi, Akif Kaynak, Mariana Paulino, Saleh Gharaie, Russell J. Varley, Abbas Z. Kouzani

**Affiliations:** 1 School of Engineering, Deakin University, Geelong, VIC 3216, Australia; pheidarian@deakin.edu.au (P.H.); akif.kaynak@deakin.edu.au (A.K.); mariana.paulino@deakin.edu.au (M.P.); s.gharaie@deakin.edu.au (S.G.); 2 Department of Wood Engineering and Technology, Gorgan University of Agricultural Sciences and Natural Resources, Gorgan 4913815739, Iran; hyousefi.ir@gmail.com; 3 Carbon Nexus at the Institute for Frontier Materials, Deakin University, Geelong, VIC 3216, Australia; russell.varley@deakin.edu.au

**Keywords:** dynamic hydrogels, nanohybrid, tannic acid, ferric ions, chitin nanofibers, self-healing, self-recovery

## Abstract

Electroconductive hydrogels with stimuli-free self-healing and self-recovery (SELF) properties and high mechanical strength for wearable strain sensors is an area of intensive research activity at the moment. Most electroconductive hydrogels, however, consist of static bonds for mechanical strength and dynamic bonds for SELF performance, presenting a challenge to improve both properties into one single hydrogel. An alternative strategy to successfully incorporate both properties into one system is via the use of stiff or rigid, yet dynamic nano-materials. In this work, a nano-hybrid modifier derived from nano-chitin coated with ferric ions and tannic acid (TA/Fe@ChNFs) is blended into a starch/polyvinyl alcohol/polyacrylic acid (St/PVA/PAA) hydrogel. It is hypothesized that the TA/Fe@ChNFs nanohybrid imparts both mechanical strength and stimuli-free SELF properties to the hydrogel via dynamic catecholato-metal coordination bonds. Additionally, the catechol groups of TA provide mussel-inspired adhesion properties to the hydrogel. Due to its electroconductivity, toughness, stimuli-free SELF properties, and self-adhesiveness, a prototype soft wearable strain sensor is created using this hydrogel and subsequently tested.

## 1. Introduction

Hydrogels are three-dimensional (3D) cross-linked hydrophilic polymers capable of maintaining a large amount of water (or any biological fluids) [1,2,3], and widely used for the fabrication of sensors [4], scaffolds [5], wound healing substrates [6], actuators [7], and so on. However, a major disadvantage is that they tend to lose functionality under load due to the long-term damage inflicted on them. To increase their lifetime, therefore, and extend potential applications, introducing self-healing and self-recovery (SELF) properties into hydrogels is a promising new approach [8]. However, the requirement for increased molecular mobility to facilitate SELF properties is in opposition to the need for increased mechanical strength because mobile crosslinks do not have enough structural integrity to keep the 3D structure of hydrogels while under load. Therefore, there is an urgent need to fabricate hydrogels that exhibit both SELF properties and adequate mechanical strength [9]. To achieve SELF properties within hydrogels, different methods have been proposed thus far, such as loading healing agents to hydrogels or incorporating dynamic covalent and/or non-covalent bonds, such as metal–ligand bonds [2,8,10,11].

Thus far, many hydrophilic polymers have been evaluated, with polysaccharides being the most promising candidates primarily because of their low cost, cytocompatibility, biocompatibility, and biodegradability [1,12]. As an example, starch in particular, is an inexpensive and readily-available polysaccharide with branched (amylopectin) and linear (amylose) polymeric forms [1,13]. However, its brittleness and moisture-sensitivity render it difficult to employ in load-bearing applications [3,12,14,15], while its lack of electroconductivity makes it equally difficult for use in soft wearable strain sensors [16,17]. Found in the exoskeleton of arthropods and synthesized linearly by glucosamine monomers with β-(1-4)-*N*-acetyl glucosamine linkages, chitin nanofibers (ChNF) is the second most abundant polysaccharide in nature and contains highly crystallized fibrous structures with diameters usually between 2–20 nm [18,19,20]. Similar to nanofibers isolated from cellulose, they have a highly modifiable surface area capable of imparting mechanical strength to hydrogels. It is hypothesized therefore that ChNFs modified with tannic acid (TA) and ferric ions (Fe^3+^) would give both SELF and mechanical properties to the hydrogel due to their stiff but dynamic structural motifs. Moreover, due to the presence of TA, the hydrogel may exhibit mussel-inspired adhesion and capable of attaching to almost any surface but still peel off without leaving any residue. This feature can make these hydrogels extremely versatile because most soft wearable strain sensors require external adhesives to eliminate interfacial delamination between the sensor and substrate, especially under repeated deformation [21,22].

A dynamic mussel-inspired hydrogel with SELF properties and high mechanical strength exhibiting electro-conductivity and self-adhesion for use in soft wearable strain sensors has been designed and synthesized in this work. To do this, a TA/Fe@ChNFs nanohybrid modifier has been synthesized and used as a modifier to a starch-based polymer network. The TA/Fe@ChNFs nanohybrid functions not only as a nanofiller but also as a dynamic cross-linking agent, providing both SELF properties and mechanical strength to the hydrogel. To date, there is little if any research available using TA/Fe@ChNFs nanohybrids to fabricate such hydrogels. Furthermore, polyvinyl alcohol (PVA) is used as a non-toxic, biodegradable, and water-soluble polymer additive to plasticize and increase the molecular mobility of the polymer. Acrylic acid (AA) monomers are grafted to the starch to increase binding affinity and enhance energy dissipation and, thus, avoid the formation of irreversible, covalent crosslinks. The SELF performance and mechanical strength of the hydrogel are enhanced by adding TA/Fe@ChNFs nanohybrid as dynamic motifs into the hydrogel.

## 2. Materials and Methods

### 2.1. Chemicals and Materials

The chitin nanofibers (ChNFs) isolated by ultrafine grinding were supplied by Nano Novin Polymer Co. (Sari, Iran). Tannic acid (TA), acrylic acid (AA), starch (St), polyvinyl alcohol (PVA), ammonium persulfate (APS), and ferric chloride hexahydrate (FeCl_3_·6H_2_O) were purchased from Sigma-Aldrich (Castle Hill, Australia). All other chemicals purchased were analytical grade unless otherwise noted and were used as received.

### 2.2. Fe/TA@ChNFs Nanohybrid Synthesis

The Fe/TA@ChNF nanohybrid was synthesized using a facile two-step procedure. In brief, 134.0 g of ChNFs suspension (1.5 wt.%, containing about 2.0 g ChNFs) was diluted using 500 cc distilled water, followed by adjusting the pH to 8.5 by dropwise addition of a tris buffer solution (1 M) into the ChNF suspension. Afterward, 2.0 g of TA was added to the suspension while stirring at ambient room temperature for 12 h. Subsequently, excess TA was washed using centrifugation and re-dispersion in distilled water to concentrate the TA-ChNFs to 3.0 wt.%. In order to introduce Fe nanoparticle into the TA-ChNFs suspension, 0.5 g of FeCl_3_·6H_2_O was dissolved in 100 mL of distilled water and stirred overnight. After that, 50.0 g of TA-ChNFs was introduced into the suspension and stirred for 6 h at ambient temperature during which time the color of the suspension changed from white to red. Finally, the suspension was centrifuged and washed with distilled water to extract the excess Fe ion solution, followed by homogeneously re-dispersing the synthesized Fe/TA@ChNFs nanohybrid in distilled water using ultrasonic treatment. The Fe/TA@ChNFs suspension was finally sealed and cryopreserved at 4 °C.

### 2.3. Fabrication of PVA-St-PAA-Fe/TA@ChNF Nanohybrid Hydrogels

The PVA-St-PAA hydrogels were synthesized based on the method developed by Hussain et al. for PVA-glycogen-PAA hydrogels [23]. Briefly, a certain content of St and PVA was dissolved in a 1:1 ratio in 100 mL distilled water at 90 °C for 2 h. After cooling at ambient temperature, 1.0 mL of APS (1.0 g/10 mL) was added to the 5 mL St-PVA solution and stirred for 10 min to activate the PVA and St functional groups. Next, an adequate amount of AA monomer was added to the solution and stirred for another 10 min to polymerize PAA. Finally, Fe/TA@ChNF nanohybrid was gradually added to the mixture at different concentrations (0.5. 1, 1.5, 2 wt.%) while stirring. To remove any formed bubbles, the mixture was sonicated for a few minutes then left for 30 min at ambient temperature to allow hydrogelation to occur. The synthesized nanocomposite hydrogels containing 0.0 wt.%, 0.5 wt.%, 1.0 wt.%, 1.5 wt.%, and 2.0 wt.% of Fe/TA@ChNF nanohybrid were identified and used throughout as follows: PVA-St-PAA-Fe/TA@ChNF0, PVA-St-PAA-Fe/TA@ChNF0.5, PVA-St-PAA-Fe/TA@ChNF1, PVA-St-PAA-Fe/TA@ChNF1.5, and PVA-St-PAA-Fe/TA@ChNF2.

### 2.4. Hydrogel Characterization

The nanostructured morphologies of ChNFs and Fe/TA@ChNFs were evaluated using transmission electron microscopy, TEM (JEOL, 2100). To do this, each sample was diluted to about 0.001 wt.% using ethanol and sonicated to prepare a well-homogenized suspension. Next, the samples were cast onto perforated carbon-coated grids and the excess liquid was absorbed using filter paper. An image analyzer program (ImageJ) was used to analyze the resulting microscopic images. The microstructured morphologies of the freeze-dried hydrogels were evaluated using scanning electron microscopy, SEM (Zeiss, Supra, Oberkochen, Germany), before and after loading Fe/TA@ChNFs. ATR-FTIR was used to analyze the ChNFs, Fe/TA-ChNFs, and hydrogel using a Bruker Vertex 70 FTIR between 600–4000 cm^−1^. Mechanical properties were determined using a universal tensile testing machine equipped with a 200 N load cell (Instron, Norwood, MA, USA). The mechanical properties of the silicone-coated rectangular-shaped specimens were determined using a constant stretching rate of 60–160 mm/min and sample dimensions of 10 mm wide, 6 mm thick, and 35 mm long. The initial distance between the two clamps was 15 mm. A visual inspection of the self-healing performance of the hydrogel was performed by cutting it in half and immediately splicing the two halves together again to form the original shape. The strength ratio between the healed and original hydrogel was defined as the healing efficiency. Rheological measurements of the hydrogels were conducted using an oscillatory rheometer equipped with a parallel plate geometry with a 40-mm diameter at a gap of 49 μm and a frequency of 1.0 Hz (TA Instruments, HR-3, New castle, DE, USA). The self-adhesive performance of the hydrogels was evaluated using different clean substrates with the same universal tensile testing machine at a crosshead speed of 10 mm/min until separation was achieved [22].

## 3. Results and Discussions

### 3.1. Design and Synthesis of PVA-St-PAA-Fe/TA@ChNF Nanohybrid Hydrogels

**Figure 1:**
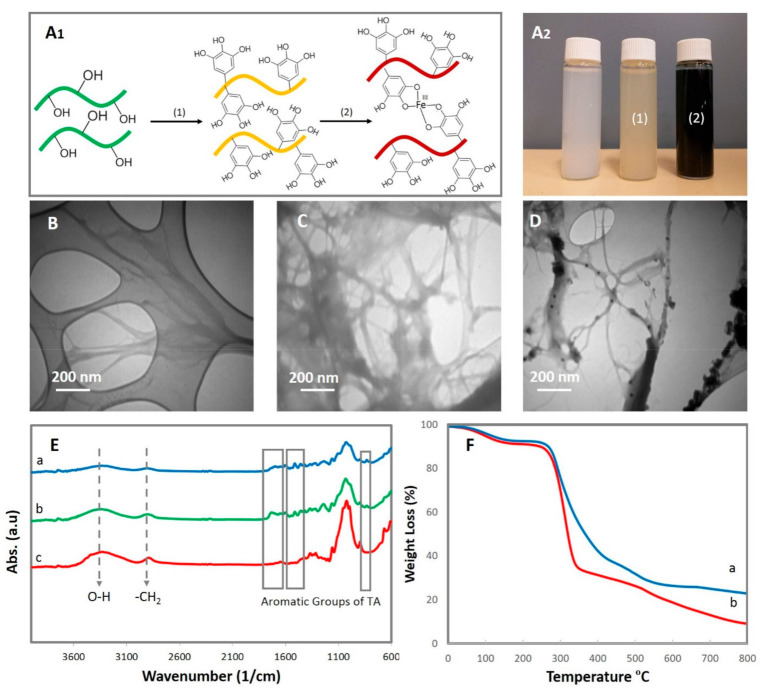
(**A_1_**) Schematic of depositing TA and Fe ions onto ChNFs and (**A_2_**) the color change of ChNFs after loading TA and Fe ions respectively, TEM image of (**B**) ChNFs, (**C**) TA-ChNFs, (**D**) Fe/TA@ChNFs, (**E**) FTIR results of (**a**) Fe/TA@ChNFs, (**b**) TA@ChNFs, (**c**) ChNFs, and (**F**) thermal stability of (**a**) Fe/TA@ChNFs and (**b**) TA@ChNFs.

In this research, a tough, self-healing, self-adhesive, and conductive hydrogel via reversible catecholato-metal coordination bonds through the incorporation of Fe/TA@ChNF nanohybrid into the PVA-St-PAA network was synthesized. To do this, TA and Fe nanoparticles were first introduced onto the surface of ChNFs, which, by virtue of its high surface area and abundance of available hydroxyl groups, was able to interact with the catechol groups within the dendritic structure of the TA (Figure 1A_1_). The oxidative polymerization of TA monomers on ChNFs under alkaline conditions changed the color of ChNFs suspension from white to yellow and introduced ample catechol groups on ChNFs that were able to adsorb Fe^3+^ ions onto the TA@ChNFs surface via chelation interactions. The presence of TA in TA@ChNFs also facilitated the in situ formation of metal nanoparticles on the TA@ChNFs surface because of its high reduction capability. Immediately after treating TA@ChNF with ferric chloride, the color of suspension changed from yellow to a dark red color (Figure 1A_2_). Based on the TEM images, the observed lengths of ChNFs and TA@ChNF were on the micrometer scale, while their diameters were on the nanoscale (62 ± 17 nm, Figure 1B and 69 ± 28 nm, Figure 1C). Successful deposition of Fe nanoparticles on ChNFs was confirmed using TEM. As seen in Figure 1D, dense but uniform Fe nanoparticles (15–25 nm) were observed due to the successful formation of Fe/TA@ChNF nanohybrid. According to the FTIR spectra (Figure 1E), the peaks at 3434 cm^−1^ in the ChNFs, TA@ChNF and Fe/TA@ChNFs spectra could be attributed to O-H stretching, while the bands in the 2800–3000 cm^−1^ region to -CH_2_ symmetrical and asymmetrical stretching vibrations of chitin. The amide I band of chitin was also observed at 1619 cm^−1^ and the CONH_2_ group of chitin was observed at 1307 cm^−1^. After polymerization of TA, stretching peaks at 813, 1531, and 1612 cm^−1^ were observed in TA@ChNFs, which are not present in pristine ChNFs and were attributed to the presence of stretching vibrations of C-C aromatic bonds. These findings complement those of similar studies on the deposition of TA onto the surface of cellulose nanocrystals [24]. A significant decrease in the intensity of the major peaks and broadening of the absorption peak at 3434 cm^−1^ for the Fe/TA@ChNFs nanohybrid was observed due to the consumption of hydroxyl groups by Fe nanoparticles. The effect of Fe nanoparticles on the thermal stability of TA@ChNFs was also studied (Figure 1F). After deposition of Fe nanoparticles, the decomposition of TA@ChNFs became slower than without Fe nanoparticles at 250 °C, which can be due to the interactions between Fe nanoparticles and TA@ChNFs.

**Figure 2:**
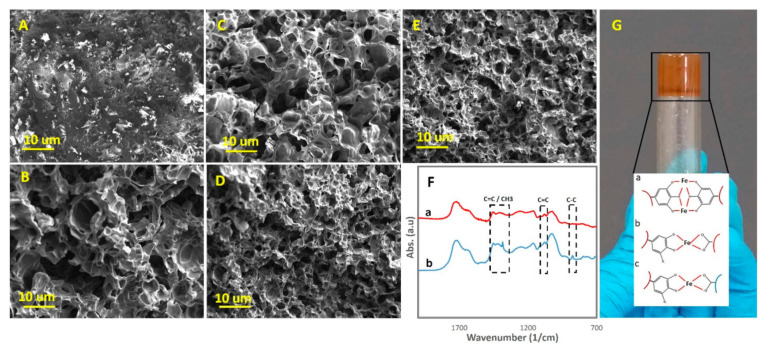
SEM images of (**A**) unreinforced hydrogel, (**B**) PVA-St-PAA-Fe/TA@ChNF0.5 (**C**), PVA-St-PAA-Fe/TA@ChNF1, (**D**) PVA-St-PAA-Fe/TA@ChNF1.5, (**E**) PVA-St-PAA-Fe/TA@ChNF2, (**F**) FTIR results of (**a**) PVA-St-PAA hydrogel and (**b**) and PVA-St-PAA-Fe/TA@ChNF0.5 hydrogel, (**G**) PVA-St-PAA-Fe/TA@ChNF0.5 hydrogel with different modes of dynamic catecholato–metal coordination bonds.

It was hypothesized that the presence of both TA and Fe nanoparticles in ChNFs would bestow strong cohesiveness in the bulk to PVA-St-PAA via reversible dynamic catecholato–metal coordination bonds. Moreover, it was expected that PVA-St-PAA would show a strong self-adhesion to different substrates via a mussel-inspired adhesion. After loading Fe/TA@ChNFs, we anticipated the formation of dynamic catecholato–metal coordination bonds between Fe/TA@ChNFs and PVA-St-PAA and ionic coordination bonding between Fe nanoparticles and carboxylic groups of PAA and hydroxyl groups of St and PVA. These coordination modes, together with hydrogen bonding between the hydroxyl groups of Fe/TA@ChNFs and the functional groups of St, PVA, and PAA, formed a strong reversible 3D dynamic network that instantly formed a hydrogel. The influence of loading Fe/TA@ChNFs on the morphology of PVA-St-PAA was studied using SEM images (Figure 2A–E). As can be seen, the fracture surface of the freeze-dried hydrogels showed a difference in morphology and pore size to that of the loaded and unloaded Fe/TA@ChNFs hydrogels. The unloaded sample showed a nonhomogeneous morphology (Figure 2A) while the Fe/TA@ChNFs loaded counterparts showed a small, dense, high surface area, and uniform pore size (Figure 2B–E). Additionally, the pore sizes decreased by increasing the Fe/TA@ChNFs concentrations in the hydrogels. Hence, the incorporation of Fe/TA@ChNFs nanohybrids had a significant effect on the structural homogeneity of PVA-St-PAA due to the formation of reversible bonds between polymers chains. Figure 2F(b) shows the FTIR spectra of the PVA-St-PAA-Fe/TA@ChNF0.5. Absorption peaks related to the C=C distortion vibration (1563 cm^−1^), CH2 shearing vibration (1422 cm^−1^), C-O stretching vibration (1051 cm^−1^), and C-C stretching vibration (809 cm^−1^) were detected, proving the successful introduction of Fe/TA@ChNFs into the PVA-St-PAA hydrogel. Figure 2G shows the 0.5 wt.% Fe/TA@ChNF-assisted PVA-St-PAA hydrogel.

### 3.2. Mechanical, Rheological, and SELF Properties

**Figure 3:**
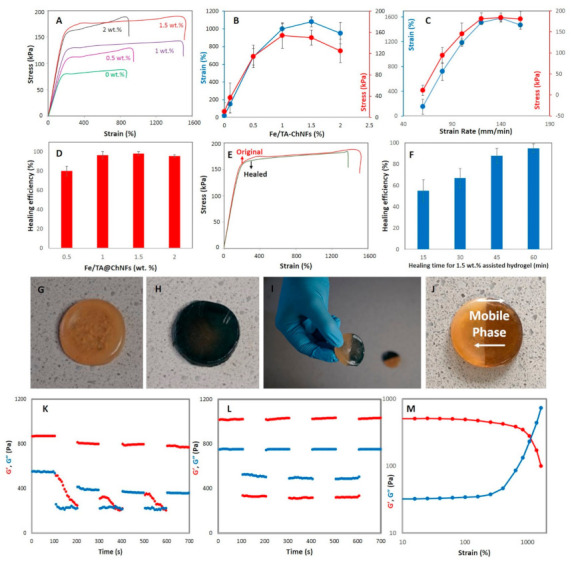
(**A**) Tensile stress–strain curves of the hydrogels at different Fe/TA@ChNF nanohybrid concentrations, (**B**) tensile tests for the notched hydrogels at different Fe/TA@ChNF nanohybrid concentrations, (**C**) tensile test of the hydrogel reinforced by 1.5 wt.% Fe/TA@ChNF nanohybrid at different strain rates, (**D**) healing efficiency of the hydrogels at different Fe/TA@ChNF nanohybrid concentrations, (**E**) typical stress–strain curves of the pristine and healed hydrogels reinforced by 1.5 wt.% Fe/TA@ChNF nanohybrid concentration, (**F**) time-dependency behavior of 1.5 wt.% Fe/TA@ChNF hydrogels during the self-healing process, (**G**–**I**) visual self-healing inspection of the 1.5 wt.% Fe/TA@ChNF hydrogel, (**J**) the healed hydrogel after 20 min, breakup and reformation ability of (**K**) unreinforced hydrogel and (**L**) 1.5 wt.% Fe/TA@ChNF hydrogel at 1 and 1000% strains and a fixed time interval, and (**M**) shear-thinning behavior of the 1.5 wt.% Fe/TA@ChNF hydrogel.

It is proposed, therefore, that inclusion of Fe/TA@ChNF nanohybrids with rigid but dynamic motifs not only improves the mechanical properties of PVA-St-PAA hydrogel but these also act as dynamic cross-linkers. Hence, they can be used instead of static cross-linkers like *N*,*N*′-methylene bisacrylamide during the polymerization of PAA. To demonstrate this, we prepared rectangular shape hydrogels followed by stretching them using a universal tensile test. The stress–strain curves of the hydrogels containing different percentages of Fe/TA@ChNF nanohybrid (0, 0.5, 1, 1.5, and 2 wt.%) are shown in Figure 3A. The stretchability, toughness, and strength of the hydrogels reached 1503%, 184.1 kPa, and 2.27 MJ/m^3^ by loading 1.5 wt.% Fe/TA@ChNF nanohybrid to the hydrogels, revealing that Fe/TA@ChNF nanohybrids can increase the mechanical properties of the hydrogels and create highly stretchable hydrogels. However, further increasing of Fe/TA@ChNF nanohybrid (2 wt.%) drastically reduced the mechanical properties of the hydrogels. This can be related to the formation of aggregations within the network of hydrogels acting as stress concentration points, thus adversely dropping the mechanical properties of hydrogel under stress. To evaluate the efficiency of the hydrogel against crack propagation while stretching, a notch was created in each hydrogel and it was observed that the Fe/TA@ChNF nanohybrid greatly improved blunting of any propagating crack with the highest improvement in toughness achieved for the 1.5 wt.% Fe/TA@ChNF nanohybrid-assisted hydrogel (Figure 3B). To track the strain-rate dependency, mechanical tests were conducted on 1.5 wt.% Fe/TA@ChNF nanohybrid-assisted hydrogel at 60–160 mm/min stretching rates. It was found that higher strain rates lead to higher breaking strain, although the strain rate dependency disappeared after 120 mm/min because of the reduction in energy dissipation efficiency of the reversible dynamic catecholato–metal coordination bonds (Figure 3C) [24]. The effect of Fe/TA@ChNF nanohybrid on self-healing of the hydrogels was then quantified using the tensile strength ratio between the healed and original hydrogels (Figure 3D). As seen, by increasing Fe/TA@ChNF nanohybrid the self-healing performance of hydrogel increased from 80% for 0.5 wt.% Fe/TA@ChNF nanohybrid-assisted hydrogel to 96.5% for 1 wt.% Fe/TA@ChNF nanohybrid-assisted hydrogel after 60 min healing time and then showed little further increase despite a maximum healing of 98% for 1.5 wt.% Fe/TA@ChNF nanohybrid-assisted hydrogel. As seen, the healed 1.5 wt.% Fe/TA@ChNF hydrogel almost recovered to its initial mechanical properties after 60 min healing (Figure 3E). To demonstrate the time-dependent self-healing of the hydrogels, self-healing was measured for the 1.5 wt.% Fe/TA@ChNF hydrogels at different time frames (20, 40, and 60 min) (Figure 3F). As seen, the healing efficiency reached 79% after 20 min healing time and then slowed down reaching 96.5% after 40 min, and finally 98% after 60 min healing.

Visual inspection of self-healing (Figure 3G–J) was performed using two 1.5 wt.% Fe/TA@ChNF nanohybrid-assisted hydrogels, one of which had been dyed with methylene blue (Figure 3H). The samples were cut in half and rejoined at the cutline using samples of different colors to clearly illustrate healing. The hydrogel showed a stimuli-free and rapid self-healing response with immediate stability and stretchability across the cutline thanks to the presence of strong dynamic bonds (Figure 3I and Video S1). Interestingly, the blue in the sample started to fade (Figure 3J) presumably a result of the molecular mobility and the dynamic covalent bonding, such as the formation of ionic and metal chelating bonds (Figure 3G). To illustrate self-healing of the reinforced and unreinforced hydrogels at the microscopic level, the breakup and reformation of the hydrogel networks using an alternate step strain test (strain = 1% and 1000%) at a fixed time interval (100 s) (Figure 3K,L) was explored. As seen, at low amplitude oscillatory shear (strain = 1%) the storage moduli (G′) of both reinforced and unreinforced hydrogels are higher than their loss moduli (G″) indicating that the 3D network of the hydrogel remains intact under small oscillatory strain over time. After subjecting the hydrogel to high amplitude oscillatory shear (strain = 1000%) over the next 100 s however, G′ and G″ were inverted meaning that the 3D hydrogel network had collapsed and transformed into a sol-state. Despite showing the formation of a hydrogel-state (G′ > G″) by returning from high strain to low strain at a fixed frequency (1.0 Hz) for the unreinforced hydrogel, it did not fully recover the disrupted 3D network of hydrogel due to lacking dynamic bonds (Figure 3K). On the other hand, an instantaneous, repeatable, and complete self-recovery of the disrupted 3D network was observed for the reinforced hydrogel after disruption indicating the excellent self-healing or recovery of the hydrogel due to the reversible bonds in the hydrogel structure (Figure 3L). To explain this, it is proposed that at a very high strain, the strong catecholato–metal coordination bonds start to unzip, causing the observed changes in G′ and G″ and transformation from a hydrogel to a sol state (Figure 3M). Therefore, by combining SELF mechanical properties, the hydrogels formed here are clearly very versatile and can potentially be employed for many practical applications.

### 3.3. Self-Adhesiveness, Electrical Conductivity, and Strain Sensing Test

**Figure 4:**
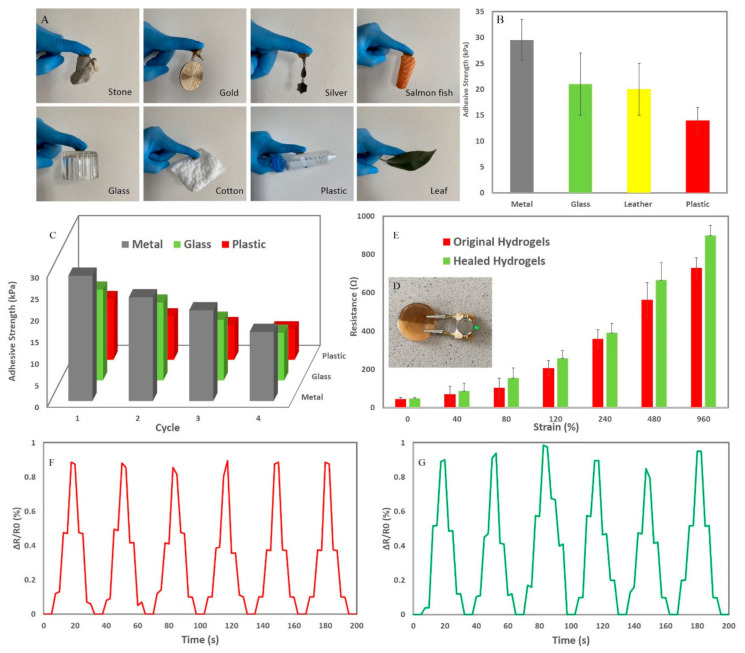
(**A**) Self-adhesiveness of the hydrogel to different substrates, (**B**) measurement of the self-adhesion strength of the hydrogels on different substrates by a tensile test, (**C**) Cyclic self-adhesiveness measurement of the hydrogel on different substrates, (**D**) electroconductivity of the cut hydrogel after healing, (**E**) the resistance of original and healed hydrogel at different strains, and monitoring the relative resistance change of (**F**) original and (**G**) healed hydrogels adhered to the first author’s forefinger to monitor its bending with different angles in real-time.

In addition to imparting SELF, toughness, and stretchability, the presence of catechol moieties of TA can also impart mussel-inspired self-adhesiveness to the hydrogel. These properties, when combined with the observed electronic properties, can make the hydrogels developed here particularly unique for soft wearable strain sensing applications [21,24,25,26]. To reveal the remarkable self-adhesive capability of the hydrogel on a wide range of substrates, the hydrogel was adhered to a range of different surfaces, including stone, gold, silver, glass, salmon tissue, plastic, cotton, and tree leaf. As seen in Figure 4A, the hydrogel was used to attach all of these substrates to a nitrile rubber glove and bore the weight of the item. To quantify this qualitative observation, a cyclic peel-off tensile test was performed four times by attaching the hydrogel onto different substrates (rubber, plastic, glass, metal, and leather) and the adhesive load during peeling was determined (Figure 4B). The hydrogel adhered to all of these surfaces while showing a repeatable self-adhesive performance with the highest adhesion strength between the hydrogel and metal interface that can be related to the existence of metal complexation at the interface of them (Figure 4C). Furthermore, π-π stacking or CH-π interaction can be considered as the mechanism of the adhesion between plastic and hydrogel. Similarly, hydrogen bonding can be deemed as the main adhesion mechanism between glass, leather, and rubber to the hydrogel [21,24]. The hydrogel was also attached to a wooden mannequin’s elbow to observe stretchability and self-adhesiveness of the hydrogel. As seen in Video S2, the hydrogel can be stretched without detaching, thus showing simultaneously self-adhesion and stretchability suggesting that this hydrogel may be suitable for soft wearable strain sensors.

Polymers like PAA can be cross-linked by ions (Fe^3+^, Ca^2+^, and Li^+^). Hence, the presence of ample cross-linked ions can impart excellent electrical conductivity and high performance in the strain sensor to hydrogels. The electrical conductivity of the hydrogel was also evaluated by simply using a light-emitting diode (LED) indicator. As seen in Video S3, the LED bulb was immediately lit after connecting the hydrogel to the electric circuit and reversibly lit and darkened by stretching and relaxing the hydrogel because of local disconnections. As seen in Figure 4D, the LED bulb was lit after connecting the healed hydrogel to the electric circuit and restored the electric circuit at the molecular level after self-healing of the hydrogel. Next, the hydrogels resistance was measured with a multimeter at a probe distance of 1 cm and stretching the hydrogel up to 960%. As seen in Figure 4E, the resistance of the hydrogel increased with applying a tension due to the increasing distance between the conductive segments of the 3D network and increasing void content within the hydrogel network [21]. As a result, the electrical conductivity of the hydrogel together with its self-healing, self-adhesiveness, toughness, and stretchability increases the versatility of the hydrogel for many practical applications, including soft wearable strain sensors. Moreover, unlike most hydrogel-based strain sensors needing an external adhesion for attaching to the body, these hydrogels do not need any external adhesion thanks to their self-adhesiveness performance. Additionally, since static crosslinkers were not used during the fabrication of the hydrogel, it is, thus, not brittle and will not easily lose its functionality while under load [24]. To detect the strains of the body, the hydrogel was used as a soft wearable strain sensor by adhering to a human forefinger. Once attached, the original (Figure 4F) and healed (Figure 4G) sensors responded well to finger strain. It was observed that the resistance increased to higher values by bending the forefinger and stretching the sensor, in agreement with the results of Figure 4E. The resistance was repeatedly recoverable to its initial value when a human forefinger was straightened while displaying an identical resistance pattern. Importantly, the initial resistance was restorable. The relative resistance change of the hydrogel after 200 s was stable, albeit with some slight fluctuations (Figure 4F). As a result, due to a stable, reproducible, and detective resistance change at different bending angles, the hydrogel was able to reliably monitor the real-time resistance displaying remarkable suitability as a soft wearable strain sensor with high mechanical strength, SELF-performance, and self-adhesion. The healed sensor was also attached to a human forefinger to evaluate the SELF-performance of the sensor under the same strains. The results showed that the healed sensor was able to successfully detect similar patterns comparable to those detected by the original hydrogel (Figure 4G). This confirms that this hydrogel, thanks to its high healing efficiency, is suitable for long-term use in soft wearable strain sensors.

## 4. Conclusions

A nano-polysaccharide mixed hydrogel with excellent SELF, mechanical, electrical, and self-adhesiveness performance suitable for soft wearable strain sensors has been presented. For this purpose, ChNFs were used as the nano-polysaccharide and were coated with ferric ions and tannic acid (TA/Fe@ChNFs) to provide the dynamic covalent structural motifs. Using a facile method, the electroconductive polysaccharide-based hydrogel was synthesized from starch/polyvinyl alcohol/polyacrylic acid (St/PVA/PAA) hydrogels, followed by loading TA/Fe@ChNFs into the hydrogel network. Due to the incorporation of TA/Fe@ChNFs, thereby forming hydrogen bonding and chelation coordination bonds, it was observed to have excellent SELF ability—without needing any external stimuli—whilst having reliable mechanical, electrical, and self-adhesion properties. Hence, this hydrogel is anticipated to be ideally suited for use in soft wearable strain sensors. For the future work, extra attention can be paid to cost, toxicity, processability, and biocompatibility while designing such hydrogels to meet industrial and application requirements. Additionally, the mechanism of self-healing at nanoscale should be assessed.

## Data Availability

Not applicable.

## Supplementary Materials

The following are available online at https://www.mdpi.com/article/10.3390/s21113574/s1, Video S1: Rapid self-healing response with immediate stability and stretchability; Video S2: Stretchability and self-adhesiveness of the hydrogel to a wooden mannequin’s elbow; Video S3: Evaluation of the electrical conductivity of the hydrogel using a light-emitting diode (LED) indicator.
